# Reliability of team-based self-monitoring in critical events: a pilot study

**DOI:** 10.1186/1471-227X-13-22

**Published:** 2013-12-01

**Authors:** Martin Stocker, Lynda Menadue, Suzan Kakat, Kumi De Costa, Julie Combes, Winston Banya, Mary Lane, Ajay Desai, Margarita Burmester

**Affiliations:** 1 NICU/PICU Children’s Hospital Lucerne, CH-6000, Lucerne 16, Switzerland; 2 Department of Anaesthesia, Singapore General Hospital, Outram Road, Singapore 169608, Singapore; 3 CICU Great Ormond Street Hospital, Great Ormond Street, WC1N 3JH, London, UK; 4 PICU Royal Brompton Hospital, Sydney Street, SW3 6NP, London, UK; 5 Epidemiology and Biostatistics, Royal Brompton Hospital, Sydney Street, SW3 6NP, London, UK; 6 Department of Anaesthesia, Royal Brompton Hospital, Sydney Street, SW3 6NP, London, UK

**Keywords:** Teamwork, Self-assessment, Mayo high performance teamwork scale, Resuscitation, Patient safety

## Abstract

**Background:**

Teamwork is a critical component during critical events. Assessment is mandatory for remediation and to target training programmes for observed performance gaps.

**Methods:**

The primary purpose was to test the feasibility of team-based self-monitoring of crisis resource management with a validated teamwork assessment tool. A secondary purpose was to assess item-specific reliability and content validity in order to develop a modified context-optimised assessment tool.

We conducted a prospective, single-centre study to assess team-based self-monitoring of teamwork after in-situ inter-professional simulated critical events by comparison with an assessment by observers. The Mayo High Performance Teamwork Scale (MHPTS) was used as the assessment tool with evaluation of internal consistency, item-specific consensus estimates for agreement between participating teams and observers, and content validity.

**Results:**

105 participants and 58 observers completed the MHPTS after a total of 16 simulated critical events over 8 months. Summative internal consistency of the MHPTS calculated as Cronbach’s alpha was acceptable with 0.712 for observers and 0.710 for participants. Overall consensus estimates for dichotomous data (agreement/non-agreement) was 0.62 (Cohen’s kappa; IQ-range 0.31-0.87). 6/16 items had excellent (kappa > 0.8) and 3/16 good reliability (kappa > 0.6). Short questions concerning easy to observe behaviours were more likely to be reliable. The MHPTS was modified using a threshold for good reliability of kappa > 0.6. The result is a 9 item self-assessment tool (TeamMonitor) with a calculated median kappa of 0.86 (IQ-range: 0.67-1.0) and good content validity.

**Conclusions:**

Team-based self-monitoring with the MHPTS to assess team performance during simulated critical events is feasible. A context-based modification of the tool is achievable with good internal consistency and content validity. Further studies are needed to investigate if team-based self-monitoring may be used as part of a programme of assessment to target training programmes for observed performance gaps.

## Background

The contribution of human factors and team-work failures to medical error and adverse patient safety is well documented. The report “To err is human: Building a safer health-care system” states that the majority of medical errors are not the result of individual failures, but defects at the team, system or process level [1]. Improving teamwork offers a route to improve patient safety and the Patient Safety First campaign advises “where appropriate, train as a team” [2]. The literature supports the effectiveness of team training, stating “Better teamwork, better performance” [3,4]. McGaghie and colleagues critically reviewed simulation based medical education research and concluded that principles for health care team training are evidence-based, and that simulation-based training is a key element [5]. Longitudinal studies reporting a beneficial impact of a team training programme in a paediatric setting have been published by the SPRinT (Simulated Paediatric Resuscitation and Team Training) programme and others [6,7].

Team performance is complex and difficult to assess. Kardong-Edgren recently reviewed 22 simulation evaluation tools and concluded most tools are not sufficiently assessed regarding reliability and validity and many are not focused on teamwork [8]. There are several specific teamwork rating scales but they differ in terms of resource requirement, need for expert raters, reliability and context validity [9-13]. A recently published review of survey instruments measuring teamwork in health care settings emphasises the importance to select and adapt one of the published instruments according to context and research question before creating a new tool [14]. In our study, the Mayo High Performance Teamwork Scale (MHPTS) was chosen because in the context of multi-professional assessment it has good reliability and validity and low resource requirements [11]. However, no scale itself is valid and validity needs to be supported in the context by 5 different entities: Content, response process, internal structure, relationship to other variables, and consequences [15].

Assessment is a critical component for feedback and remediation, which is mandatory for the learning and changes in behaviour that can lead to improved patient safety [16]. Van Der Vleuten’s conceptual framework of programmatic assessment argues that a deliberate set of longitudinal assessments is superior to single or individual data and that aggregated assessment points are the best basis for a reasonable assessment with effective impact for learning [17]. Using this framework, this study is the first step to developing a programmatic assessment (longitudinal assessment in simulated and real critical events) of teamwork at our institution. Participant self-monitoring is the only achievable way to receive assessments in real critical events with low costs and resources. Self-assessment in our study is used in the view of the new conception of self-monitoring reported recently by Eva [18]. This concept characterises self-monitoring as a prompt, context-based assessment of specific behaviours.

The primary aim of our study was to assess the feasibility of team-based self-monitoring using the MHPTS after simulated critical events. The secondary aim was to evaluate the item specific agreement of the MHPTS between the team and observers. Where unsatisfactory item specific agreement was identified we aimed to adapt the MHPTS to our context and to assess content validity of the modified tool. This is in accordance with other groups using modified versions of the MHPTS [19,20]. Reliability and feasibility with a handy, easy to use scale are important aspects of team-based self-monitoring. This study serves as pilot trial developing and evaluating a longitudinal assessment programme of multidisciplinary teamwork at our institution.

## Methods

### Study setting and participants

This was a prospective, single-centre study carried out from December 2010 to August 2011 on the Paediatric Intensive Care Unit (PICU) of a specialist cardio-respiratory hospital in the UK (Royal Brompton Hospital, London). In-situ embedded SPRinT courses were performed every 2 weeks by an interprofessional faculty that always included at least one nurse (PICU or paediatric) and one doctor (PICU consultant/fellow or Anaesthetic consultant). All faculty members had received UK and US training in simulation and adult learning specifically with reference to crisis resource management and debriefing techniques. Course participants (always at least 4 members) were interprofessional and included nurses, cardiologists, intensivists, anaesthetists, surgeons and allied health professionals working in paediatrics and on PICU. These courses consisted of didactic crisis resource management and team training, a high fidelity simulated critical event scenario, and video assisted debriefing. Simulated scenarios were derived from real events to obtain clinically relevant, realistic scenarios. All scenarios were conducted with a high fidelity mannequin (SimBaby, Laerdal^**©**^) in a dedicated PICU bed space which was set up according to local protocols. Participants were asked to provide care as realistically as possible acting on physiological variables from the mannequin and the monitor. Airway management, cardiopulmonary resuscitation including defibrillation, echocardiography, insertion of intravenous catheters, drawing up and administration of medications (with the exception of controlled medications) were part of the scenario.

### Crisis resource management (CRM) and assessment

The SPRinT programme is primarily focused on 4 CRM principles. The principles taught are derived and adapted from those identified as key to improving team performance in paediatric critical care [21,22], anaesthesia [23], and multi-professional cardiac arrest teams [24]. Role clarity (leader, specific roles), communication (closed loop communication, transmission of frequent plans, addressing people directly, maintaining good tone), resources awareness and utilization (unit resources, personnel support, knowledge of the hospital emergency system) and situational awareness (global assessment, avoiding fixation, error prevention) are the key features of the training. The MHPTS provides a representative sample of these key behaviours for efficient and effective teamwork [11]. Within the MHPTS all items (questions) are scored according to a graded scale (0 = never, 1 = inconsistently, 2 = consistently) or marked not applicable (NA). Participants and trained observers (2 or more SPRinT faculty) used the MHPTS to assess team performance immediately after each scenario. Agreement between participants (self-assessment) and observers (objective assessment) was measured for all 16 items.

### Statistical analysis

Internal consistency of the MHPTS was reported with Cronbach’s alpha. The summative data was reported separately for the group of observers and participants. An alpha of > 0.7 was set as the limit for acceptable reliability [25]. Consensus estimates of single items between observers and participants were reported with Cohen’s kappa analysis. A kappa > 0.8 was assessed as excellent, > 0.6 as good reliability. Item specific median group scores for each item were compared between observer and participant groups for each SPRinT course. Scores were dichotomized in agreement and non-agreement, where agreement was defined as median +/− 0.5. When items had a majority not applicable (NA) or missing answer by observer or participant group, they were scored as “not applicable”. There is a broad discussion regarding the use of median or mean for analysis in Likert type scales [26,27]. We analysed our data in the traditional approach with non-parametric procedures for ordinal scales [26]. Since this method can be considered a conservative method of analysis [27], consensus estimates were also calculated using parametric tests as control analysis (detailed data not shown).

Written consent of all participants was obtained and presented data was anonymised with no risk of identification. Questionnaires were a standard part of the educational SPRinT programme and as such did not require ethical approval according to the ethical guidelines of the British Educational Research Association (BERA) [28]. The study has not been previously published. All authors had full access to study data and take responsibility for the integrity and accuracy of data analysis. There were no competing interests and no funding for the study.

## Results

105 participants consisting of 41 physicians, 61 nurses and 3 allied health professionals, and 58 trained observers completed the MHPTS after a total of 16 SPRinT courses from December 2010 to August 2011. Each scenario had 4 to 9 participants (median 7) and 2 to 8 observers (median 4). 48 participants had never attended a SPRinT course before; 27 had attended 1 or 2 courses; 21 had attended 3 to 5, and 8 had attended more than 5 scenarios (1 unknown). A total of 2608 scores were analysed (1680 from participants, 928 from observers). Summative internal consistency of the MHPTS calculated as Cronbach’s alpha was acceptable (> 0.7) with 0.712 for the group of observers and 0.710 for the team.

The 2608 scores resulted in 256 paired scores (16 items of the MHPTS over 16 scenarios) for calculation of agreement between observers and participants. 47 scores out of 256 (18%) were marked as “not applicable”. Non-parametric analysis with Cohen’s kappa showed consensus estimates for dichotomized data (agreement/non-agreement) with good reliability (median kappa 0.62) for all matched questions together (Interquartile range (IQR) 0.31 – 0.87). As a control, parametric analysis with Cohen’s kappa for agreement showed excellent reliability (median kappa of 0.85, IQR: 0.53 – 1.0).

We chose the non-parametric analysis of item specific consensus estimates with a threshold for good reliability of kappa > 0.6 to modify the original MHPTS. Item-specific analysis revealed 7 questions with poor reliability (kappa < 0.6) which were abandoned; these were either with longer and more complex sentences (question 12 and 15), difficult to observe behaviours (questions 7, 8, 11, 13, 16) or items regarding errors and complications (questions 12, 13, 15). There were 6 matched questions with excellent reliability (kappa > 0.8: Questions 1, 3, 5, 9, 10 and 14) and 3 with good reliability (kappa > 0.6: Questions 2, 4 and 6) (Table 1). Two of the items with excellent reliability showed a high percentage of not applicable scores: question 9 was “not applicable” in 75% (12 out of 16) of courses and question 14 in 56% (9 out of 16 courses).

**Table 1:** Item-specific consensus estimates between the group of observers and participants

	**Questions (items) of the Mayo high performance teamworks scale**	**Kappa-value**	**Agreement**	**Not applicable**
1	Do you feel that the leader was recognized by all team members?	0.872	Very good	0
2	Do you think the leader assured maintenance of an appropriate balance between command authority and team member participation?	0.625	Good	0
3	Do you feel that each team member demonstrated clear understanding of his/her role?	0.862	Very good	0
4	Do you think team members prompted each other to attend to all significant clinical indicators throughout the patient?	0.714	Good	0
5	Do you think team members verbalized their activities loud when they were actively involved with the patient?	1.	Perfect	0
6	Do you think team members repeated back for paraphrased instructions and clarifications to indicate that they heard them correctly?	0.614	Good	0
7	Do you think team members referred to established protocols and checklists for the procedure/invention?	0.294	Fair	0
8	Do you feel that all members of the team were appropriately involved and participated in the activity?	0.259	Fair	0
9	Do you feel that disagreement of conflicts among team members were addressed without a loss of situation awareness?	1.0	Perfect	75%
10	Do you think roles were shifted to address urgent or emergent events when appropriate?	0.846	Very good	0
11	Do you think team members acknowledged their lack of understanding and ask for repetition clarification when directions were unclear?	0.333	Fair	19%
12	Do you feel that team members acknowledged-in a positive manner-statements directed at avoiding or containing errors or seeking clarification?	0.111	Poor	50%
13	Do you think team member called attention to actions that they felt could cause of complications?	0.111	Poor	50%
14	Do you think team members responded to potential errors or complications with procedures that avoided the error of complication?	1.0	Perfect	56%
15	Do you think team members persisted seeking a response when statements directed at avoiding or containing errors or complications did not elicit a response or contain the error?	0	None	63%
16	Do you think team members asked each other for assistance prior to or during periods of task overload?	0.4	Fair	6%

These 9 questions formed a new self-monitoring tool (TeamMonitor: Table 2) with a resulting median kappa of 0.86 (IQR: 0.67 - 1.0). The content validity of TeamMonitor was then examined with reference to the 4 key CRM principles of the SPRinT programme (blueprint examination). Every principle is mapped at least 3 times. Role clarity is mapped to questions 1, 2, 3, and 8 (recognition of the leader, team member participation with clear understanding of roles, and shifting role when appropriate). Communication is mapped to questions 2, 5, and 6(maintenance of appropriate command authority of the leader, verbalizing activities and repeating back or paraphrasing instructions and clarifications). Resource awareness and utilization is mapped to questions 3, 4, and 8 (understanding team members’ roles, prompting each other to attend to significant indicators and shifting roles when appropriate). Situational awareness is mapped to questions 4, 7, and 9 (conflicts among team members without loss of situation awareness, avoiding the potential errors and instruction within the team to attend to all significant clinical indicators).

**Table 2:** Teamwork self-assessment tool: TeamMonitor (modified mayo high performance teamwork scale)

	**Use the following scale to rate the team on each dimension:**	**0 = never/rarely**	**1 = inconsistently**	**2 = consistently**	**na = not applicable**			
1	Do you feel that the leader was recognized by all team members?				0	1	2	na
2	Do you think the leader assured maintenance of an appropriate balance between command authority and team member participation?				0	1	2	na
3	Do you feel that each team member prompted each other to attend to all significant clinical indicators throughout the scenario?				0	1	2	na
4	Do you think team members prompted each other to attend to all significant clinical indicators throughout the scenario?				0	1	2	na
5	Do you think team members verbalized their activities loud when they were actively involved with the patient?				0	1	2	na
6	Do you feel that team members repeated back or paraphrased instructions and clarifications to indicate that they heard them correctly?				0	1	2	na
7	Do you feel that disagreement or conflicts among team members were addressed without a loss situation awareness?				0	1	2	na
8	Do you think roles were shifted to address urgent or emergent events when appropriate?				0	1	2	na
9	Do you think team members responded to potential errors or complications with procedures that avoided the error or complication?				0	1	2	na

Comments: Please rate conservatively: Most teams that have not worked extensively together do not demonstrate many of the qualities.

## Discussion

Team-based self-monitoring of teamwork in simulated critical events is feasible. The original MHPTS showed an acceptable internal consistency (alpha = 0.71) in our study without a significant difference between observers and team participants of the SPRinT training programme. Our results show a lower reported internal consistency compared to Malec at 0.85 [11]. However, those scenarios were designed with intended CRM problems (i.e. fixation error, distraction) whilst our study used scenarios derived from real critical untoward events without introduction of created CRM problems. It is possible that scenarios targeted to negative teamwork events contain some bias and facilitate rating of obvious CRM problems. Our aim is to have a reliable self-assessment tool for real events, therefore we believe it is reasonable to use scenarios derived from real events that are so realistic that they will themselves stimulate real and relevant CRM problems. Simulations that recreate the real clinical environment delivering an authentic learning experience have been shown to improve the effectiveness of interprofessional education and crucially, enhance the transferability of learning from simulated to real clinical encounters [29,30]. Malec reported a high inter-rater agreement of participant ratings without special training to use the original MHPTS [11]. In our context with a self-monitoring assessment it is crucial to have a concise, comprehensible and easy to use assessment tool.

Analysis of item specific agreement between the team and observers in our study showed a reasonable reliability (kappa = 0.62) with a wide range. Malec reported a good item specific inter-rater reliability, whereas Hamilton reported a reliability of 0.64 using the original MHPTS for rating team behaviour during trauma resuscitation which is similar to our study [11,19]. We found that questions with good agreement are shorter, clearer and easy to observe. Therefore, how a question is phrased may be an important factor for reliable self-monitoring. On the other hand, a question with low reliability may not demonstrate a defective item, but the possibility that the scenario did not have the capability to elicit a clear response.

Our modified self-assessment tool TeamMonitor has a high reliability (kappa = 0.86). Hamilton modified the original MHPTS as well and piloted his prototypical team-scoring instrument for trauma resuscitation [19]. Interestingly, he ended up with a modified MHPTS of 7 items and 5 of them correspond to our modified self-assessment tool TeamMonitor (questions 1, 3, 4, 5, 6). The study by Hamilton has the same limitation as the study by Malec of scenario selection bias representing a spectrum from ineffective to effective team behaviour. In our study, questions concerning situational awareness, errors and complications have a high percentage of answers rated “not applicable” (questions 9, 13, 14, 15) which is in agreement with other studies [11,31,32]. It is possible that these items were not understood by the learners or that our scenarios did not challenge participants in these areas. The importance of situational awareness can be difficult to determine and this factor may be more prominent in the clinical context of real events [31,32]. Items concerning errors could have been infrequently answered due to emotional barriers or a lack of self-awareness. Despite the current positive tendency to reduce individual culpability in relation to the importance of systemic factors, physicians and nurses should be encouraged to be aware of individual errors and barriers in clinical practice [33]. Therefore, since it is important to have questions mapping situational awareness, errors and complications, we included questions 9 and 14 as they had perfect consensus agreement. We tested content validity by comparing CRM principles for effective teamwork with the 9 items of TeamMonitor and found a good representation.

There is ongoing debate regarding reliability of self-assessments with differing views as to whether physicians are able to accurately self-assess or not [34,35]. Studies have demonstrated that physicians can reliably self-assess competence, but when it comes to self-evaluation for performance (applying personally determined standards) the result is unsatisfactory [35]. It may be that despite some limitations, self-assessment remains an essential tool as guidance for self-reflection. Recently, Eva reported a new conception of self-assessment ability [18]. In the past, most studies regarding self-assessment were carried out asking “guess your grade” [36]. This question refers to a global statement of one’s ability relative to other people. Eva’s new conceptual framework makes a distinction between global self-assessment as a cumulative judgement based on an unguided review of one′s experience and self-assessment as a process of self-monitoring in the moment [18,37]. Global self-assessment has been shown to be poor [34]. Results of self-monitoring as a situation-specific self-awareness are much more accurate [18,37]. Our team-based self-assessment is very similar to the conceptualised process of self-monitoring according to Eva: i) the assessment is in the context of a performance, ii) all items are asking regarding situational awareness for specific behaviours and iii) there is no rating comparing one’s own performance with peers. In addition, in order to minimize individual bias and outliers due to personal factors or lack of situational awareness, individual scores were transformed into team scores. We accordingly named our assessment process “team-based self-monitoring”.

There are limitations to this study that need further research and evaluation. All validity is construct validity with multiple sources [15]. We only tested internal structure and content validity. We did not examine response process, relationship to other variables and consequences. Nevertheless, our study serves as pilot trial and the first step in developing and evaluating a longitudinal assessment programme of multidisciplinary teamwork at our institution. Response process, discrimination validity and consequences as target training programmes for observed performance gaps need to be evaluated during implementation of the adapted assessment tool TeamMonitor. No assessment scale itself is valid and our results are specific to the context of the interprofessional SPRinT training programme. Justification of items concerning situational awareness and errors that had a high percentage response of “not applicable” requires a factor analysis carried out with a larger sample. In addition future studies are needed to investigate whether the instrument is reliable in the clinical context of real events and whether our findings are generalizable to other environments and specialities.

## Conclusions

Team-based self-monitoring with the MHPTS to assess team performance during simulated critical events is feasible, with increased reliability for short questions regarding easy to observed behaviours. A context-based modification of the tool, TeamMonitor, is achievable with good internal consistency and content validity. Whether TeamMonitor can be used to target team training programmes for identified performance gaps needs to be further evaluated.

## Competing interests

All authors declare that none author have support from a company for the submitted work; no authors have relationships with companies that might have an interest in the submitted work; their spouses, partners, or children have no financial relationships that may be relevant to the submitted work; and no authors have non-financial interests that may be relevant to the submitted work.

## Authors’ contributions

SM was responsible for the design of the study, data acquisition and analysis and the final manuscript; ML helped in the design of the study, participated in the study and made substantial contributions to the manuscript; KS participated in the study and was responsible for data acquisition; DK participated in the study, helped for data acquisition and made contributions to the manuscript; CJ participated in the study, helped for data acquisition and made contributions to the manuscript; BW was responsible for statistical data analysis and interpretation; LM participated in the study and made substantial contributions to the manuscript; DA participated in the study and made substantial contributions to the manuscript; BM helped in the design of the study, participated in the study and made substantial contributions to the manuscript. All authors have read and approved the final manuscript. All authors have substantially contributed to preparing the manuscript and no other person has contributed significantly to the manuscript.

## Pre-publication history

The pre-publication history for this paper can be accessed here:

http://www.biomedcentral.com/1471-227X/13/22/prepub
